# Application-specific approaches to MicroCT for evaluation of mouse models of pulmonary disease

**DOI:** 10.1371/journal.pone.0281452

**Published:** 2023-02-09

**Authors:** Elizabeth F. Redente, Katrina W. Kopf, Ali N. Bahadur, Annette Robichaud, Lennart K. Lundblad, Lindsay T. McDonald

**Affiliations:** 1 Department of Pediatrics, National Jewish Health, Denver, Colorado, United States of America; 2 Department of Medicine, University of Colorado Anschutz Medical Campus, Aurora, Colorado, United States of America; 3 Department of Academic Affairs, National Jewish Health, Denver, Colorado, United States of America; 4 Bruker BioSpin Corporation, Billerica, Massachusetts, United States of America; 5 SCIREQ Inc., Montreal, Quebec, Canada; 6 Department of Medicine, McGill University, Montreal, Quebec, Canada; 7 Ralph H. Johnson VA Medical Center, Charleston, South Carolina, United States of America; Ohio State University, UNITED STATES

## Abstract

The advent of micro-computed tomography (microCT) has provided significant advancement in our ability to generate clinically relevant assessments of lung health and disease in small animal models. As microCT use to generate outcomes analysis in pulmonary preclinical models has increased there have been substantial improvements in image quality and resolution, and data analysis software. However, there are limited published methods for standardized imaging and automated analysis available for investigators. Manual quantitative analysis of microCT images is complicated by the presence of inflammation and parenchymal disease. To improve the efficiency and limit user-associated bias, we have developed an automated pulmonary air and tissue segmentation (PATS) task list to segment lung air volume and lung tissue volume for quantitative analysis. We demonstrate the effective use of the PATS task list using four distinct methods for imaging, 1) *in vivo* respiration controlled scanning using a *flexi*Vent, 2) longitudinal breath-gated *in vivo* scanning in resolving and non-resolving pulmonary disease initiated by lipopolysaccharide-, bleomycin-, and silica-exposure, 3) post-mortem imaging, and 4) *ex vivo* high-resolution scanning. The accuracy of the PATS task list was compared to manual segmentation. The use of these imaging techniques and automated quantification methodology across multiple models of lung injury and fibrosis demonstrates the broad applicability and adaptability of microCT to various lung diseases and small animal models and presents a significant advance in efficiency and standardization of preclinical microCT imaging and analysis for the field of pulmonary research.

## Introduction

Micro computed tomography (microCT) is a state-of-the-art tool that can provide a highly relevant preclinical assessment of pulmonary disease. MicroCT has been previously been shown to accurately correlate with pulmonary physiology and histological features in several disease models [1–5]. Thus, microCT has become a valuable addition to a broad variety of laboratory based pulmonary research outcomes, particularly with the ability to image animals longitudinally throughout a study capturing more than end-of-study data. Technological advancements have improved the utility of microCT for basic and translational animal studies, allowing for increased resolution of images, improved scanning efficiency and reduced radiation exposure for live and longitudinal imaging [6–11]. However, several challenges for animal imaging remain, including 1) a lack of standardized imaging methodologies that can be applied across disease pathology [2, 12]; 2) reduced resolution of images due to respiratory and cardiac movement artifact [13, 14]; 3) a limited ability to automate segmentation of tissue volume fractions with currently available analysis platforms [12] and 4) a lack of a standardized and automated analysis pipeline between laboratories. In particular, these challenges have broadly limited the ability to generate reproducible and quantitative parameters for the often heterogenous disease patterns that are characteristic of animal models which develop a patchy distribution of lung disease [15]. In 2017, a workshop report on the Use of Animal Models for the Preclinical Assessment of Potential Therapies for Pulmonary Fibrosis concluded there was not sufficient evidence to recommend microCT scanning for preclinical assessment of antifibrotic therapy as a routine methodology [15]. As the need for rapidly translatable preclinical data increases, the importance of addressing these challenges and developing and adopting standardized methodologies becomes critical [16].

Implementing standardized approaches accelerates the acquisition of accurate, reproducible, highly sensitive and dynamic preclinical data in rodents. These techniques can be applied to multiple models of lung disease development and progression, such as tumor studies, lung transplantation, lung development models, exposure-induced models and in longitudinal studies involving therapeutic intervention. Several novel techniques for image acquisition and analysis have been recently published [1, 2, 17–19], however, a standardized method has yet to be established, and there are currently limited published resources for investigators to determine appropriate imaging and analysis approaches to meet study requirements. Herein, we describe four approaches to microCT with detailed methodological information and discussion of advantages and limitations of each approach. While newer image-based gating strategies [10] have significantly improved the ability to obtain high-resolution reconstructions, movement artifact in live-animal scans still limits image resolution. To address this concern, we present alternative, novel post-mortem and *ex vivo* imaging techniques to generate high resolution images and reconstructions. Further, while aerated lung volumes are readily segmented using standardized automation, quantitative tissue parameters have been limited by an inability for standard analysis programs to automatically segment the lung tissue volume fraction, leaving investigators to rely on time-intensive and error prone manual segmentation techniques.

Thus, microCT data have largely provided qualitative assessment of images with a limited ability to directly quantitate changes in diseased/inflamed/remodeled tissue and parenchymal architecture. In models where disease has historically been defined as an increase in lung tissue volume relative to aerated lung volume using histological analysis methods [20], without the ability to segment diseased or accumulated inflammation within tissue, compensatory changes in lung volume may obscure important tissue:volume ratios occurring with disease progression [12]. Recent studies using manually segmented lungs have shown decreased aerated lung volume during peak fibrosis in the bleomycin model of pulmonary fibrosis [21], whereas the silica-induced model of PF resulted in increased aerated lung volume [2]. Automated segmentation of the tissue volume fraction has been challenging since heart tissue, vasculature, and the diaphragm are similar in density to fibrotic tissue, malignant masses, and inflammatory infiltrates [11]. Therefore, we sought to develop an automated task list for pulmonary air and tissue segmentation (PATS) using three models of pulmonary injury and disease. The PATS task list has overcome the need for manual segmentation and lack of reproducibility across laboratories by providing a method for automated segmentation of the air volume as a total region-of-interest (ROI), and subsequent analysis of the tissue parameters within the air-ROI. Through this method, the efficiency of quantifying tissue volume, air volume, and changes in the pulmonary microarchitecture is substantially improved, while the automated approach reduces bias and error. The ability to reconstruct high-resolution scans of *in vivo*, intact post-mortem, and *ex vivo* lung tissue has provided a unique opportunity to assess the whole lung, thereby overcoming the challenge of heterogeneity of disease and sampling that occurs using standard post-mortem histological methods [16, 22, 23]. These approaches address major challenges in the field including application of a standardized and automated analysis pipeline which can be applied to multiple disease models, providing a quantitative analysis of air and tissue volumes, high quality image resolution, whole lung assessment, and three-dimensional (3D) reconstruction capabilities.

## Materials and methods

### Animals

Male C57BL6/J mice (Stock #00064, Jackson Laboratories, Bar Harbor, ME) 8–10 weeks of age, 20–30 g bodyweight. Animals were provided with food and water ad libitum, routine husbandry, standard housing, and 12-hour light/dark cycle.

### Models of lung disease and injury

To induce pulmonary fibrosis, mice were anesthetized with isoflurane (Baxter Pharmaceuticals) and were intratracheally instilled with silica (0.2g/kg) in sterile saline (0.9%, 60 μl), as described previously [24], or bleomycin (50 μl, 1.5U/kg, Amneal Biosciences) [22, 25]. Control animals received saline (0.9%, 60 μl) or were naïve. To induce acute lung injury, 20 μg LPS (E. coli 055:B5, Sigma Aldrich, St Louis, MO) in sterile saline (0.9%, 50 μl), was administered intratracheally [26].

### Micro-computed tomography

MicroCT images were obtained on a Bruker Skyscan 1176 at the Ralph H. Johnson VA Medical Center or on a Skyscan 1276 at National Jewish Heatlh (indicated in figure legends, Bruker MicroCT, Kontich, Belgium). Longitudinal and, respiration-controlled studies images were acquired at 35 μm and post-mortem studies images were acquired at 9 μm resolution, with a 0.5 mm Aluminum filter, and 0.7° rotation step. Scans were reconstructed using NRecon software (Bruker, v1.17.7). Analysis and generation of VOIs was performed using CTAn software (Bruker) or by Amira-Avizo software (Thermo Fisher Scientific, version 2022.1) as indicated in the text. CTVol software (Bruker) was used to create 3D surface renderings from surface model files. Method-specific task lists and considerations are provided (S1 Methods).

### Longitudinal studies

Animals were maintained under isoflurane (3–5%) and were imaged in supine position. Image-based respiratory gating was used to sort images at exhalation to minimize movement artifact in the final reconstruction [10, 27]. Individual animals were tracked over time and data for multiple animals were averaged at each time point. Aerated lung volume was quantified at exhalation using image-based sorting. n = 3/group for LPS and n = 5/group for bleomycin and silica.

### Respiration-controlled

Animals were anesthetized with ketamine (100 mg/kg)/xylazine (15 mg/kg)/Acepromazine (10 mg/kg) by intraperitoneal injection, and an 18G catheter was inserted into the trachea via surgical cutdown. Animals were connected to a *flexi*Vent FX-2 (SCIREQ, Inc., Montreal, Quebec, Canada) and were mechanically ventilated using a mouse default profile (109 breaths/min, 10 mL/kg, 3 cmH_2_O PEEP [28]. Pancuronium (1 mg/kg) was administered to inhibit spontaneous breathing efforts. Flexiware software (v8.1, SCIREQ, Inc.) was integrated with the Bruker Skyscan 1276 to deliver 0.2 second breath holds in time-sequence for triggered image acquisition [2]. n = 5/group (naïve and bleomycin).

### Post-mortem nitrogen-inflation

Anesthetized mice were mechanically ventilated using the *flexi*Vent, as described above. A lethal dose of ketamine (400 mg/kg)/xylazine (50 mg/kg) was administered via intraperitoneal injection. When the heart rate declined as detected via EKG (Physiosuite, Kent Scientific, CT, USA), nitrogen gas was delivered via the *flexi*Vent in-port and ventilation was continued for 1–3 minutes at 3 cmH_2_O PEEP [17, 29, 30]. A deep lung inflation to 30 cmH_2_O was performed over 3 seconds and the trachea tied off at maximum inflation. Following rigor mortis, animals were imaged by microCT. n = 4/group naïve and 6/group silica.

### *Ex vivo* preparation and imaging

Lungs of euthanized, exsanguinated mice were inflated with 4% paraformaldehyde at 25 cmH_2_O and fixed in 4% paraformaldehyde overnight at 4°C. Lungs were chemically dried [31]. As an alternative method, lungs can be air inflated to a pressure of 20 cm H_2_O, perfusion fixed and dried at 30°C for 3 days [22, 32]. Scans were reconstructed into cross-sectional images with an isotropic voxel size of 9 μm. MicroCT acquisition parameters were: X-ray tube voltage 50 kV, current 500 μA, exposure time: 900 ms with 0.5 mm AI filter and a 0.3° rotation step. n = 4/group naïve and 6/group silica.

### Manual ROI generation

Scans from three naïve and three bleomycin-treated lungs (3 wks post exposure) were used to generate manual ROI’s from two independent investigators. Using the CTAn software, ROIs were manually drawn on every other image throughout the entire lung. Manual segmentation took an average of 60 minutes per mouse when performed by a veterinarian or a researcher with expertise in small animal imaging.

### Pulmonary Air and Tissue Segmentation (PATS) task list

The automated steps of the PATS task list are provided and the function of each operation is detailed (Table 1). Gray highlight indicates automated steps for aerated lung volume segmentation. The full task list is required for automated segmentation of vessel and lung tissue ROI. Optional Steps(s) are recommended for samples requiring additional modification to obtain accurate ROI. * Indicates parameters where modification may be required or beneficial depending on individual investigator needs and reconstruction/image settings. Automated segmentation took an average of 10 minutes per mouse when performed by a veterinarian or a researcher with expertise in small animal imaging.

**Table 1:** PATS task list of operations. Operations listed use terminology for CTAn software (Bruker). Similar operations are found in other analysis software. For Amira-Avizo software (ThermoFisher): Remove island command in Amira = despeckle operation in CTAn. The lung volume was calculated with the material statistics command in Amira. Grow and shrink volume commands in Amira = morphological operation in CTAn.

Operation	Parameters	Result
Thresholding	Global 55–255*	Segments body from air in image
ROI shrink-wrap	2D space, stretch over holes, diameter 4 pixels*	Defines ROI as body
Reload	Image	Reloads image within the body ROI
Thresholding	Global 0–55*	Segments air in image
Bitwise Operations	ROI = Image and ROI	Defines the ROI as the image within the ROI (ROI = air within the body)
Despeckle	Sweep, 3D space, all except the largest object*, ROI	Removes outside objects included in ROI
OPTIONAL STEP Despeckle	Sweep, 3D space, all except the largest object*, ROI	Removes outside objects such as less dense body tissue included in ROI
*Comment*	*The following model and data are for aerated lung*	*Annotates data file for reference*
Save Bitmaps	ROI, BMP, Aerated lung ROI, convert to monochrome, copy shadow projection, copy dataset log file	Saves ROI created from the task list
3D Analysis	Basic values, save file as aerated lung	Analyzes parameters based on Aerated lung ROI
3D Model	.stl, ROI, binary, marching cubes 33, voxel	Builds and saves 3D model file for automated aerated lung ROI
Morphological Operations	Closing, 2D space, Round, 40*, ROI	Fills-in holes in aerated lung ROI
OPTIONAL STEP Bitwise Operations	ROI = Image and ROI	Sets ROI to image inside ROI
OPTIONAL STEP Save Bitmaps	ROI, BMP, save as ROI for modification, convert to monochrome, copy shadow projection, copy dataset log file	Saves ROI for manual modification–useful for more severe models, or models with peripheral tissue/inflammation
Reload	Image	Reloads image
Thresholding	Global 55–255*	Segments tissue within ROI
Morphological Operations	Erosion, 2D space, round, radius 1, apply to image	Erodes peripheral tissue (Note: some tissue within lung may be removed with this step)
Bitwise Operations	ROI = ROI and image	Sets ROI to image inside ROI
*Comment*	*The following data and model are for vessels and dense tissue*	*Annotates data file for reference*
Save Bitmaps	ROI, BMP, Automated ROI for lung tissue, convert to monochrome, copy shadow projection, copy dataset log file	Saves ROI created from the task list
3D Analysis	Basic values, Additional values (structure thickness, structure linear density), save as tissue volume results	Analyzes parameter based on automated lung vessels and dense tissue ROI
3D Model	.stl, ROI, binary, marching cubes 33, voxel	Builds and saves 3D model file for automated lung vessels and dense tissue ROI

### Statistics

Statistical analysis was performed using GraphPad Prism9 (GraphPad Software, San Diego, CA). The mean ±SEM are presented for longitudinal data. Unpaired two-tailed *t*-test with Welch’s correction was used to compare groups with p≤0.05 considered significant. The Pearson correlation coefficient (r) and coefficient of determination (R^2^) were determined between every dataset pair. Experiments have been designed to provide >80% power to detect an effect size of 1.6 with a Gaussian distribution (2-sided t-test, p<0.05).

### Ethics

The data generated did include human samples and therefore did not require IRB approval. All experiments involving live animals were carried out in accordance with the National Institutes of Health Guide for Care and Use of Laboratory Animals and were reviewed and received written approval by the Institutional Animal Care and Use Committee (IACUC)s at the Ralph H. Johnson VAMC, Charleston, SC, or National Jewish Health, Denver, Colorado.

## Results

### Respiration-controlled imaging and automated task list assessment

**Fig 1:**
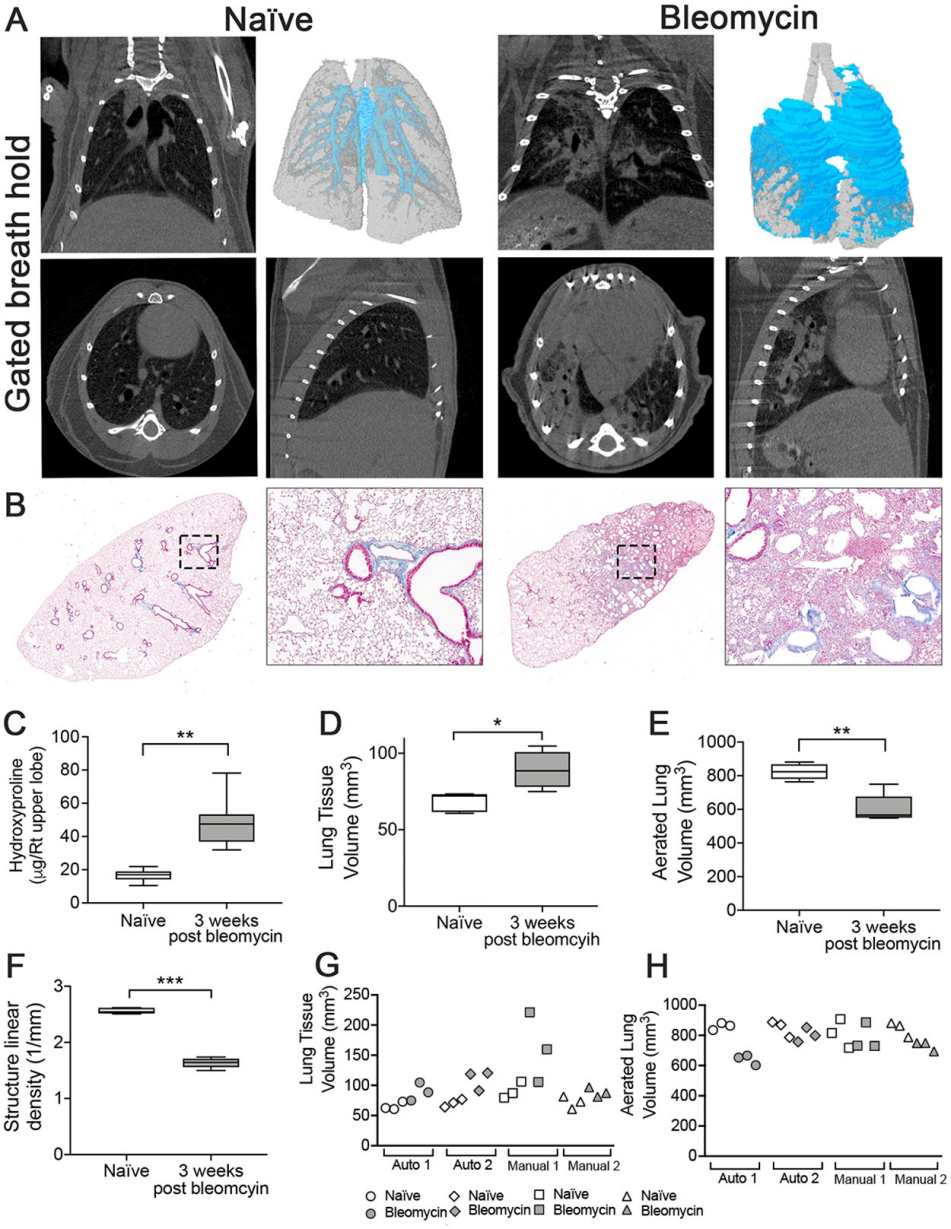
Respiration-gated imaging in bleomycin induced fibrosis. (A) microCT images from a respiration-gated scan of a saline and a bleomycin-instilled mouse 3 weeks post-instillation imaged on a Bruker Skyscan 1276 at 35 μm resolution. Representative images of coronal (top left), transverse (bottom left), and sagittal (bottom right) slices and a 3D surface rendering of aerated (gray) and tissue (blue) volume overlay (top right) are shown. (B) Representative images of lung tissue sections stained with Masson’s Trichrome (Magnification 2X (left) and 20X (right). (C) Hydroxyproline content in lungs. PATS task list quantification of (D) lung tissue volume (E) aerated lung volume and (F) structure linear density. Scatterplots of comparing (G) lung tissue volumes and (H) aerated lung volumes calculated from automated ROI generation (Auto 1 = CTAn (Bruker) and Auto 2 = Amira-Avizo (ThermoFisher) compared ROIs generated manually by two individuals. Open symbols = naïve lung values. Closed symbols = bleomycin lung values. n = 5 mice/group. Graphed as box and whisker plot (min, max with mean). *p<0.*05*, **p<0.*01*, ***p<0.*001*, 2-tailed t-test with Welch’s correction.

Naïve and bleomycin- instilled lungs (3 weeks post-instillation) were imaged using respiration-controlled, *flexi*Vent-triggered acquisition (Fig 1A and S1B Fig). Density-based 3D reconstruction of aerated lung (gray) and lung tissue (blue) volume allows for visualization of fibrotic changes versus control (Fig 1A). Histological sections stained with Masson’s Trichrome (Fig 1B) show substantial fibrosis indicated by collagen deposition and loss of normal lung architecture. Increased hydroxyproline content also demonstrated significant fibrotic disease (Fig 1C). Segmentation and quantitation of the aerated lung and tissue volumes using the PATS task list (Table 1) demonstrated an increased tissue volume within the lungs at 3 weeks post-bleomycin (Fig 1D) and decreased aerated lung volume (Fig 1E). Additionally, quantitation of structure linear density, demonstrated a decrease in structure complexity, indicative of the loss of normal lung architecture (Fig 1F). Structure linear density is a parameter that defines the number of structures that cross a vector, providing a 3D quantitation of the parenchymal microarchitecture. To compare tissue and aerated lung volumes calculated from ROIs generated between the automated PATS task list using CTAn (Bruker) and Amira-Avizo (ThermoFisher) software and those generated by manual segmentation, two independent investigators experienced in microCT and small animal anatomy, generated ROI from the scans of three naïve and three bleomycin-treated mice (Fig 1G and 1H and Table 2).

**Table 2:** Comparison of aerated and lung tissue volume between automated and manual ROI generation.

**Aerated Lung Volume (mm** ^**3**^ **)**	**Mean ±SEM**				**p value**
	**Automated CTAn**	**Automated Amira**	**Manual 1**	**Manual 2**	
**Naïve**	860 ± 13.44	847.8 ± 31.00	813.5 ± 54.69	844 ± 28.92	ns between groups
**Bleomycin**	604.4 ± 19.38	802.2 ± 27.29*	782 ± 52.61*	730.7 ± 18.26	*p<0.01 compared to CTAn
**Tissue Lung Volume (mm** ^**3**^ **)**	**Mean ±SEM**				**p value**
	**Automated CTAn**	**Automated Amira**	**Manual 1**	**Manual 2**	
**Naïve**	65.43 ± 3.845	70.77 ± 3.66	90.99 ± 7.893	71.72 ± 6.006	ns between groups
**Bleomycin**	89.37 ± 8.599	110.1 ± 9.49	162.1 ± 33.34	88.38 ± 2.547	ns between groups

Automated ROI’s were generated using CTAn (Burker) and Amira-Avizo (ThermoFisher). n = 3 mice/group.

*p<0.*05*,

**p<0.*01* 2-tailed t-test with Welch’s correction between groups.

**Fig 2:**
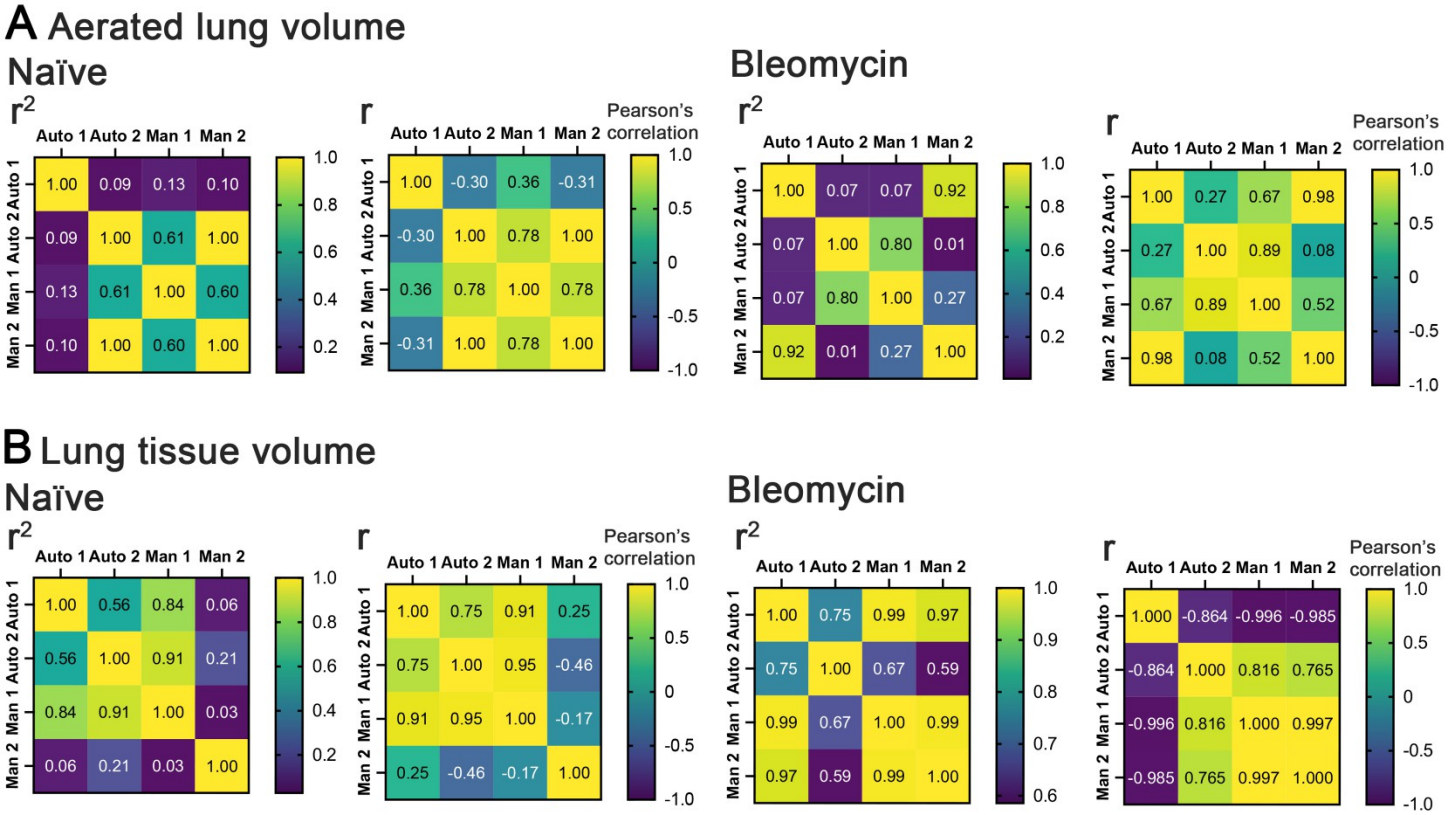
Correlation analysis between automated and manually generated ROIs. Respiration-gated Imaging in bleomycin induced fibrosis. Heat maps of: (A) aerated lung volume comparisons by R^2^ and R (Pearson’s correlation) for naïve and bleomycin-treated mice. (B) Lung tissue volume comparisons by R^2^ and R (Pearson’s correlation) for naïve and bleomycin-treated mice. Auto 1 = CTAn (Bruker) and Auto 2 = Amira-Avizo (ThermoFisher) automated generation of ROI. Man1 and Man2 = two independent generations of manual ROIs.

R^2^ and Pearson’s correlation coefficient values (r) were generated. (Fig 2A and 2B). For both the aerated lung volumes and tissue lung volumes, there was variable but overall moderate to strong correlations (r) between the automated values and manual scorers with r^2^ values covering a significant range based on manual scorer and the absence/presence of disease (Fig 2B). Overall, this indicates significant variably between the users and the manual generation of ROIs when compared to the ROIs generated from the PATS task list.

### Validation of PATS task list in multiple models of pulmonary Injury through longitudinal imaging

**Fig 3:**
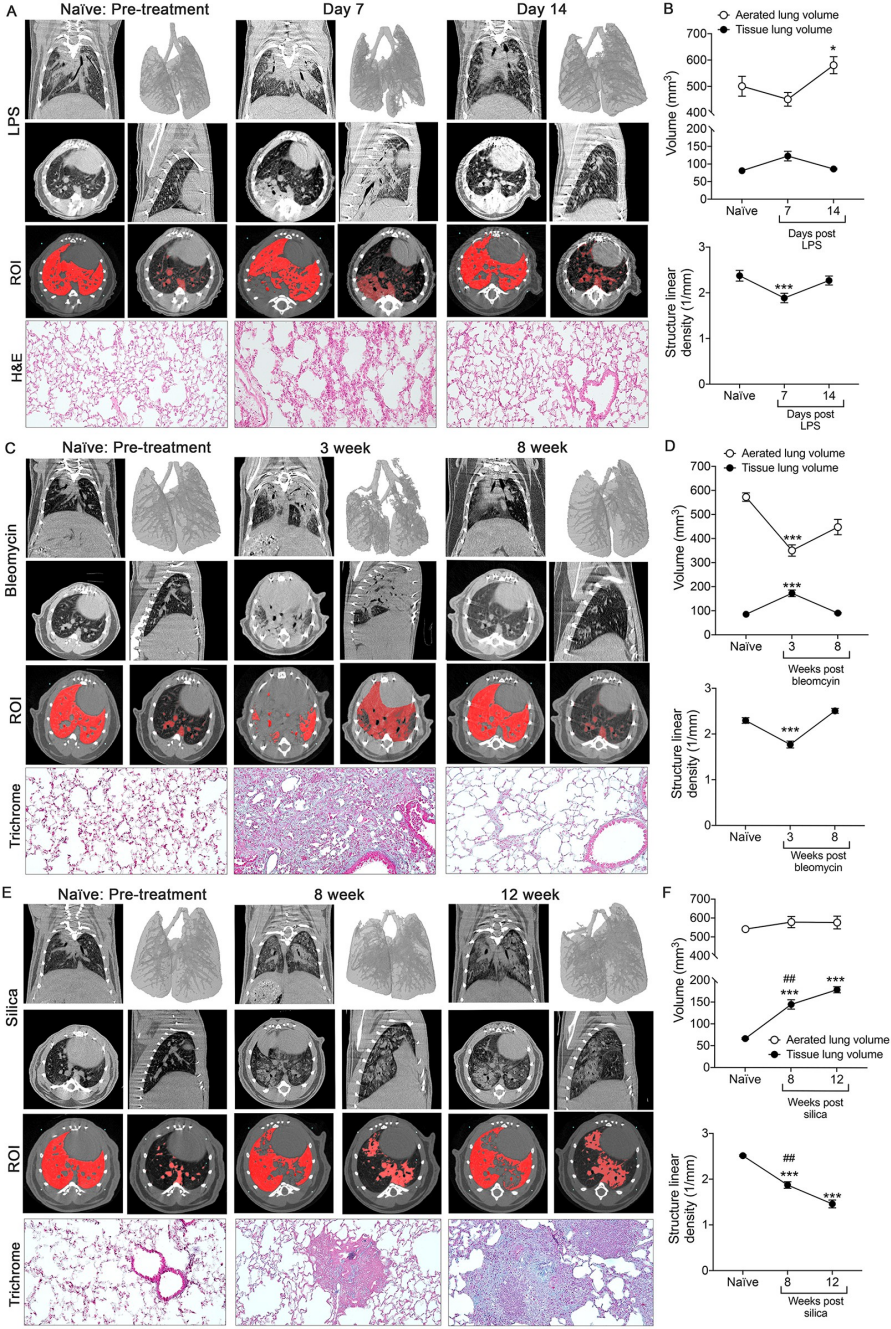
Longitudinal Imaging during the development, resolution and progression of lung disease. For longitudinal studies, mice were scanned on a Bruker Skyscan 1276 at 35 μm resolution. (A) Representative image of naïve mice scanned prior to instillation with LPS (left panels, pre-treatment), 7 days post-instillation (middle panels), and 14 days post-instillation (right panels). Images include coronal (top left), transverse (middle left), and sagittal (middle right) slices and a 3D surface rendering of aerated (gray) and tissue (blue) volume overlay (top right). PATS generated ROI for aerated lung (red, left) and lung tissue (red, right) are shown. Representative H&E images (Total Magnification 20X). (n = 3 mice/group). (B) Quantification of the aerated lung volume, tissue lung volume and structure linear density using the PATS task list. (C) Representative images of naïve mice scanned prior to instillation with bleomycin (left panels, pre-treatment), 3 weeks post-instillation (middle panels), and 8 weeks post-instillation (right panels). Images include coronal (top left), transverse (middle left), and sagittal (middle right) slices and a 3D surface rendering of aerated (gray) and tissue (blue) volume overlay (top right). PATS generated ROI for aerated lung (red, left) and lung tissue (red, right) are shown. Representative Masson’s Trichrome images (Total Magnification 20X). (n = 5 mice/group). (D) Quantification of the aerated lung volume, tissue lung volume and structure linear density using the PATS task list. (E) Representative images of naïve mice scanned prior to instillation with silica (left panels, pre-treatment), 8 weeks post-instillation (middle panels), and 12 weeks post-instillation (right panels). Images include coronal (top left), transverse (middle left), and sagittal (middle right) slices and a 3D surface rendering of aerated (gray) and tissue (blue) volume overlay (top right). PATS generated ROI for aerated lung (red, left) and lung tissue (red, right) are shown. Representative Masson’s Trichrome images (Total Magnification 20X). (n = 5 mice/group). (F) Quantification of aerated and lung tissue volumes, tissue lung volume and structure linear density using the PATS task list. *p<0.05, **p<0.01, ***p<0.001, ##p<0.01 compared to 8-week data. Open circles = aerated lung volume. Close circles = lung tissue volume. Graphed mean±SEM. *p<0.*05*, **p<0.*01*, ***p<0.*001*, 2-tailed t-test with Welch’s correction.

Longitudinal assessments of three models of lung injury; LPS, bleomycin, and silica, were performed using the PATS automated task list (Table 1, S1A–S1C Fig). Disease progression and resolution were examined for LPS and bleomycin, pre-treatment (day 0) and at maximal disease (7 days post-instillation for LPS, and 3 weeks for bleomycin), and at an endpoint following resolution (day 14 for LPS, and 8 weeks for bleomycin). Silica treated mice were imaged after 8 and 12 weeks in this progressive model. 3D rendered images from defined 3D volumes of interest (VOI) of LPS-instilled lungs demonstrated increased lung opacity indicative of inflammation at 7 days, followed by resolution at day 14 (Fig 3A). To confirm the accuracy of the PATS task list generated regions of interest (ROI) used to quantify these parameters, the air volume ROI (red, left) and lung tissue (red, right) volume ROI were each overlayed with the original microCT images (Fig 3A). Quantification indicated a significant increase in aerated volume at endpoint, a non-significant increase in tissue volume at day 7, and a significant decrease in structure linear density at day 7 returning to baseline at endpoint (Fig 3B). Bleomycin-instilled lungs exhibited a significant decrease in aerated lung volume and structure linear density with a significant increase in lung tissue volume at 3 weeks followed by resolution at 8 weeks post-instillation (Fig 3C and 3D). The increased tissue volume corresponding with the development of tissue fibrosis detected by Masson’s Trichrome staining (Fig 3C). Three-dimensional rendering and quantitation of aerated lung volume at each timepoint allows for visualization of longitudinal changes in lung capacity (Fig 3C). Silica-instilled lungs also exhibited a decrease in structure linear density and a significant increase in tissue density at 8 and 12 weeks corresponding to the development of silicotic nodules identified by Masson’s Trichrome staining (Fig 3E and 3F). However, there was not a reduction in aerated lung volume (Fig 3F). This aligns with a previous study by Dekoster et al., which also demonstrated an increase in both aerated lung and tissue volumes due to increased airway size after silica [2].

### Application of PATS task list to multiple microCT imaging methods

#### Post-mortem nitrogen-inflation imaging

**Fig 4:**
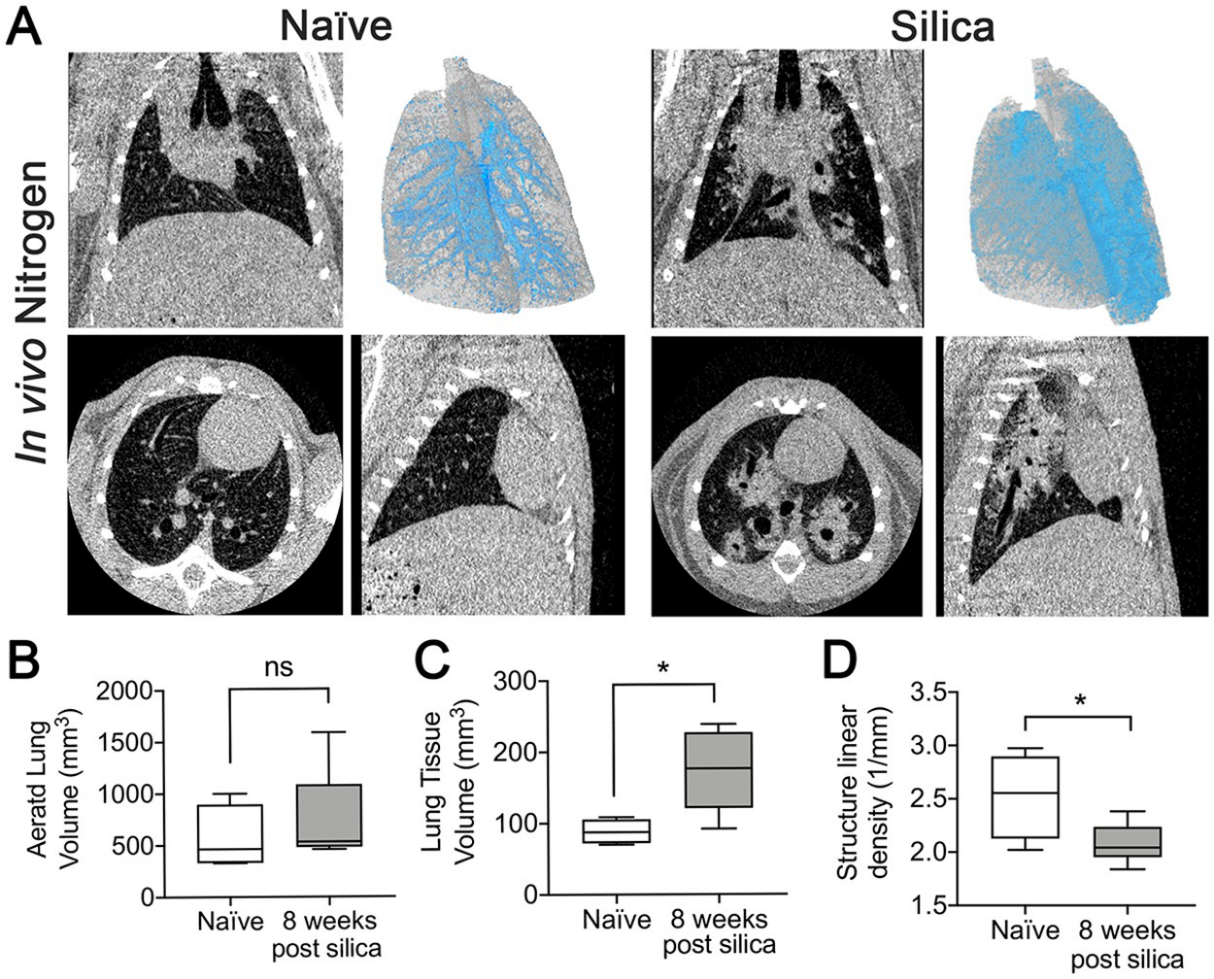
Post-mortem imaging after nitrogen inflation. (A) microCT images obtained using a Bruker Skyscan 1176 of the lungs of naïve (n = 4) or silica-instilled mice (n = 6) following nitrogen inflation. Representative images of coronal (top left), transverse (bottom left), and sagittal (bottom right) slices and a 3D surface rendering of aerated (gray) and tissue (blue) volume overlay (top right). Quantification of the (B) aerated lung volume, (C) lung tissue volume and (D) structure linear density are provided using the PATS task list. Graphed as box and whisker plot (min, max with mean). *p<0.*05*, ns = not significant. 2-tailed t-test with Welch’s correction.

Intact nitrogen-inflated lungs of naive and silica-instilled animals were assessed after 8 weeks (Fig 4A, S2 Fig). Aerated lung and tissue VOIs were generated and analyzed using the PATS automated task list (Table 1). Representative 3D model overlay of aerated (gray) and tissue volume (blue) shows increased volume of dense tissue in silica-instilled lungs (Fig 4A). Quantitative 3D analysis showed a non-significant increase in aerated lung volume in silica-instilled, versus naïve lungs (Fig 4B). Lung tissue volume was significantly increased in silica-instilled lungs (Fig 4C) and structure linear density was significantly decreased (Fig 3D), indicative of fibrotic deposition. Overlay of PATS task list generated ROI’s verifying accurate segmentation of pulmonary air and tissue volumes (S2 Fig).

#### *Ex vivo* imaging

**Fig 5:**
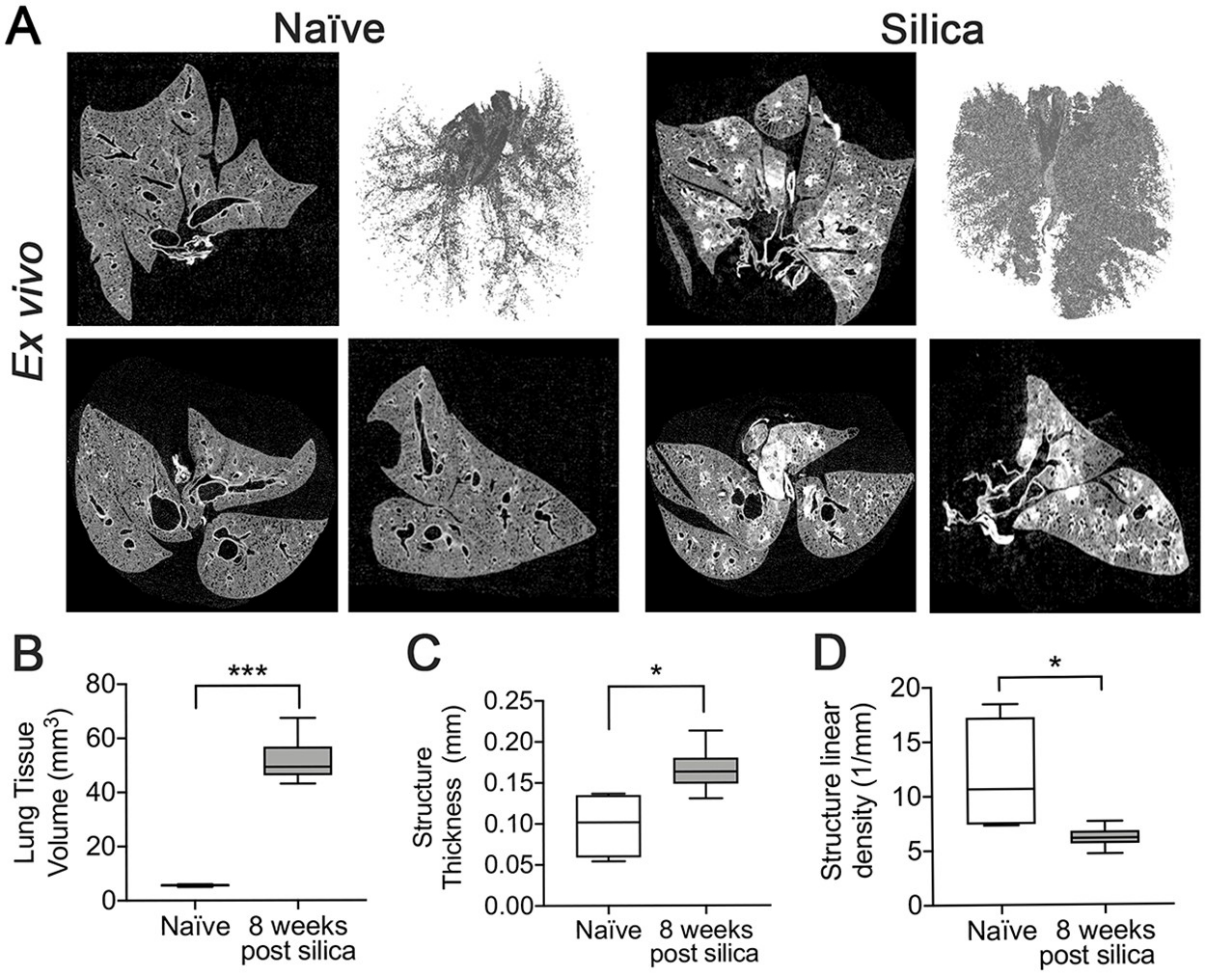
Post-mortem imaging of fixed *ex vivo* lungs. (A) microCT images from chemically dried lungs scanned using a Bruker Skyscan 1176 at 9 μm resolution from naïve mice (left panels) (n = 4), and mice 8 weeks post silica-instillation (right panels) (n = 6). Representative images of coronal (top left), transverse (bottom left), and sagittal (bottom right) slices and a 3D surface rendering of tissue volume (top right) are shown. Quantification of (B) structure thickness, (C) lung tissue volume and (D) structure linear density were generated using the *ex vivo* task list. Graphed as box and whisker plot (min, max with mean). *p<0.*05*, ***p<0.*001*, 2-tailed t-test with Welch’s correction.

Silica-instilled lungs and naïve lungs were harvested and chemically dried for high resolution microCT imaging. Silica-instilled lungs showed increased opacity (Fig 5A, S1C Fig), and increased dense tissue in 3D reconstructions (Fig 5A). Quantification of tissue parameters from a VOI containing the lung indicated significantly increased structure thickness (Fig 5B), increased lung tissue volume (Fig 5C) and significantly decreased structure linear density (Fig 5D).

## Discussion

**Fig 6:**
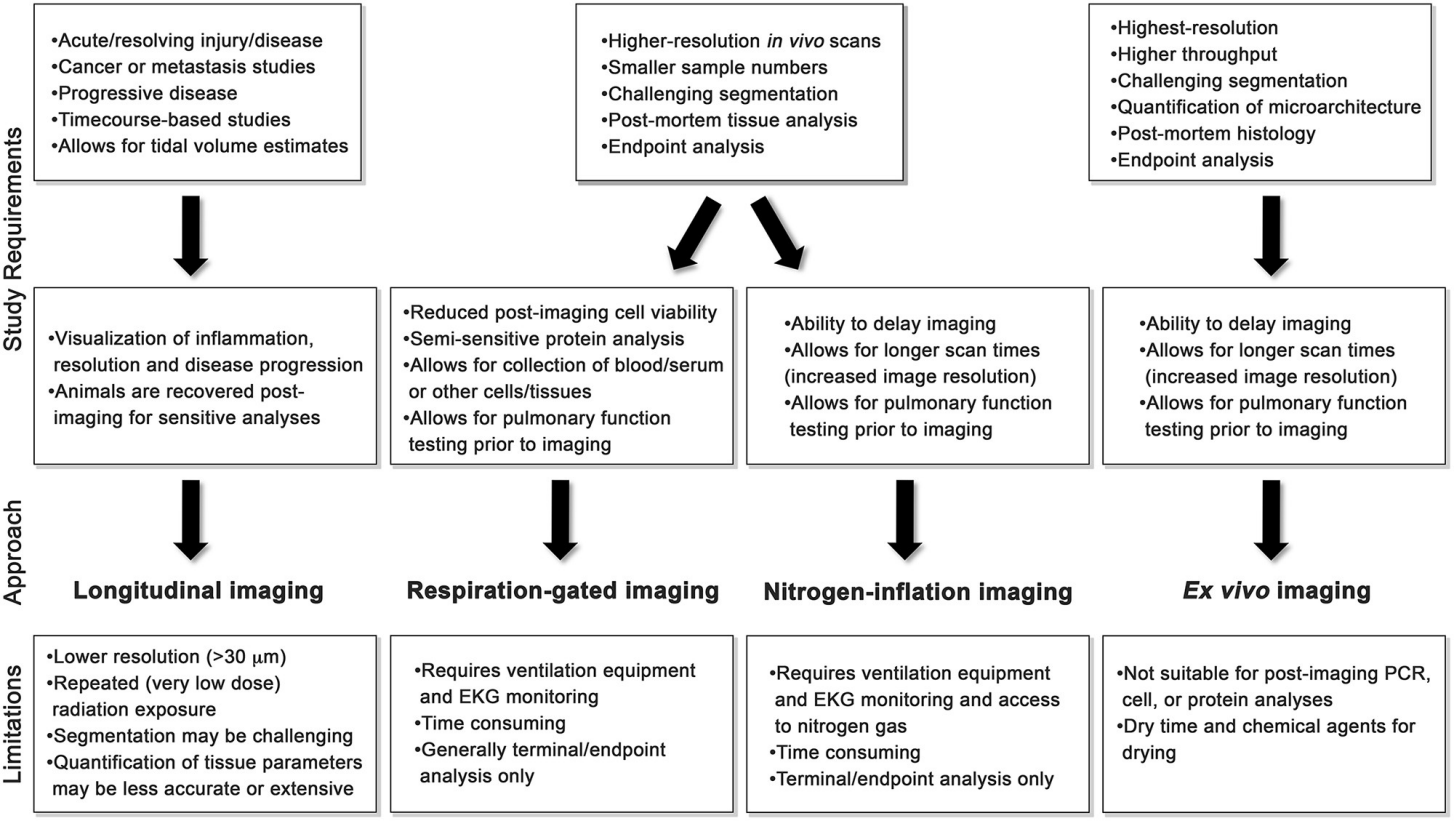
Summary and considerations of imaging modalities. A flow chart of assessment for study requirements to determine the appropriate imaging approach and associated limitations for: longitudinal-, respiration-gated-, nitrogen-inflation- and *ex vivo*-imaging.

Integration of microCT imaging into the analysis workflow for disease monitoring in small animal models is a powerful tool. It provides high quality reproducible quantitative data, can be conducted on live animals in a longitudinal manner to follow disease progression and during therapeutic intervention and generates clinically relevant outcomes. Despite these advantages, microCT imaging is often underutilized due to inherent challenges in quantitation of tissue parameters in diseased lungs and lack of published standardized techniques for live animal or fixed-tissue imaging thus requiring expert knowledge of technical CT methodology including respiration gating techniques and image thresholding as well as expertise in the interpretation of CT images. While methodologies for the reduction of movement artifact have been published [14, 33, 34], standardized methods for advanced and automated air:lung segmentation are not available. Therefore, we sought to develop an adaptable automated task list (PATS task list) and apply this to defined imaging techniques and parameters to overcome these challenges creating a set of standardized methodologies for investigators to select an application specific approach to microCT imaging for automated quantitative analysis (Table 1 and Fig 6). The PATS task list was developed using CTAn software (Bruker), and validated with the Amira-Avizo software (ThermoFisher) and the operations described are comparable across microCT software platforms. Therefore, the PATS task list represents a valuable starting point for analysis on alternative software programs. The PATS task list allows users to analyze and quantify lung tissue volumes and lung microarchitecture in addition to the standard aerated lung volumes typically reported [16]. Given the complexity of air:tissue volume ratios in animal models of pulmonary pathologies [2, 21], the ability to automate quantification of tissue parameters represents a significant improvement over previous analysis methods. Automation limits user-based variability and labor-intensive manual segmentation providing precise, reproducible and rapid data quantitation (Fig 2).

Using the PATS task list, the data presented demonstrate significant changes in aerated lung volume, tissue volume, and complexity of the parenchymal microarchitecture (structure linear density) in both longitudinal and endpoint analyses. The changes demonstrated by PATS quantification followed classic disease patterns associated with the 1) acute inflammation that defines the LPS model of lung injury, 2) resolving nature of single dose bleomycin injury and fibrosis and 3) the progressive and non-resolving nature of the silica-induced model of pulmonary. The quantitative data generated by the PATS task list was further validated by histology and hydroxyproline assay in the bleomycin model. Additionally, the accuracy of the PATS task list was validated across four methods of imaging by overlay of air and tissue regions of interest with original microCT images. For these studies, the PATS task list was applied to data collected by two different laboratories, using two different microCT imagers and analysis software programs and compared to manual segmentation. Data generated by manual segmentation, was not significantly different for lung tissue volume than that generated by the automated PATS task list, but more had more variance suggesting no loss of data integrity versus the current standard (Table 2). Given the paucity of methods to quantify lung tissue volumes *in vivo*, it is challenging to fully verify the accuracy of the tissue volumes obtained by microCT quantitation (Fig 2) and significant variability between individual users and manual ROI generation can be anticipated. This remains a limitation whether ROIs are quantified by manual or automated segmentation. However, automation allows for a standardization of analysis that can be applied cross-laboratories and across studies.

While the development of the PATS task list is a significant advancement in microCT analysis, some limitations remain. There may be cases where it is difficult to segment tissue volume from the chest wall, heart and diaphragm [35]. This can occur if there is substantial hyperattenuation due to inflammation or advanced fibrosis, during severe fibrotic disease when lung tissue lung cannot be readily distinguished from the chest wall in all images and in animals that have a large amount of subcutaneous fat. In cases like this, a rough pre-selection of an ROI can be manually done followed by the automated PATS task list. In our experience, the additional recommended steps for challenging data sets provided within the S1 Methods, addressed this challenge in most cases. A simple overlay within the analysis software of the tissue ROI with the original images or aerated volume with the original images is sufficient determine the accuracy of the automated segmentation. Enhancing image resolution through the alternative methodologies presented also improves the efficacy of the PATS task list for quantification.

Longitudinal imaging may be preferable to assess lung structure changes in acute lung injury, such as LPS or infection-induced injury and during fibrosis, emphysema or tumor development [36–43]. Images with ~30–40 μm resolution can be rapidly obtained with very low dose radiation exposure (average scan <500 mGy) [7, 11] thereby allowing live animals to be followed as a single cohort. This provides a distinct advantage for therapeutic intervention studies where pre- and post-treatment scans can be conducted on single animals and images can used for verification for the inclusion or exclusion of animals from a given study prior to treatment for improved efficiency and accuracy [2, 12, 16, 44]. In models with incomplete disease penetrance, the ability to pre-determine cohorts greatly reduces the number of animals needed to obtain statistical differences [45]. While this method provides images of sufficient quality for quantification of air volume parameters, and for visualization of processes such as inflammation, resolution, and fibrotic progression, quantification of more sensitive tissue-based parameters such as structure thickness and structure separation are best obtained through alternative microCT strategies that allow for acquisition of high-resolution images (see Fig 6 for additional considerations) [22, 23, 34, 46].

Increased resolution can be obtained in live animals using respiration-gated imaging wherein the microCT is coupled with mechanical ventilation and software trigger to control breathing and obtain images at full inspiration (breath hold) [34] however, this is often only performed as a terminal study. An advantage of this method is the ability to collect pulmonary function parameters prior to imaging. Live animal longitudinal and respiration-controlled imaging are advantageous over post-mortem or *ex vivo* approaches in that they allow for an estimation of total lung capacity while maintaining the ability to perform subsequent analysis of lung cells and tissue.

In instances where high-resolution imaging of intact lungs is required (<30 μm), post-mortem imaging may be preferable. Post-mortem *ex vivo* imaging provides superior quantification of lung parameters and is best suited to studies where endpoint assessment is required or where small differences in disease are anticipated (e.g. tumor size, metastatic area, fibrotic deposition) [22, 23, 47, 48]. An additional method involves the inflation of lungs with nitrogen gas [17]. Because the lungs do not efficiently absorb nitrogen gas, this technique can be used to obtain high resolution images at full inspiration capacity without movement artifact, providing the ability to quantify total lung volume [17]. We found that this methodology provided efficient quantification of both air and tissue parameters of the whole lung through use of the PATS task list. Similar to respiration-gated imaging, pulmonary function parameters can be obtained prior to inflation of the lungs with nitrogen gas, and tissue can be fixed and processed for histological examination or other post-mortem analyses. However, sensitive studies such as RNA analysis or primary cell isolation for culture may not be preferable due to the longer imaging times required. *Ex vivo* imaging of lung tissue provides a unique opportunity over basic histology to rapidly examine the whole lung in near histological level high resolution images which is necessary in heterogeneous disease models where even distribution of disease is not anticipated, for example in models of silicosis, or lung metastasis studies [5, 37, 42, 48–50]. In addition, the tissue is preserved for further histological analysis if special stains, or cellular level analyses are required [32]. Disadvantages include dry time and use of chemicals for drying, which prohibits downstream analyses such as PCR, flow cytometric analyses, and other assays that require live tissue.

There are many factors and considerations that shape the imaging method selected for a study. A standardized automated methodology to generate reproducible and quantitative data has been lacking in the field. Therefore, the development and validation of the PATS task list for automated segmentation of air and lung tissue volumes represents a major technical advance in the ability to apply state-of-the-art microCT technology to preclinical assessment of pulmonary disease. Implementation of standardized techniques will allow for cross-study validation and more rapid advancement of research across laboratories. Comprehensive, objective and quantifiable preclinical assessment of lung health and disease, is essential for the effective translation of basic science to patient-related metrics and outcomes. Improving efficiency of analysis, reducing bias, and expanding the number of quantifiable parameters that can be obtained from preclinical imaging through application of standardized methodologies, such as those made possible by the use of the PATS task list, are critical toward improving the utility of microCT for preclinical studies of lung health, disease and therapeutic intervention.

## Supporting information

S1 Fig Schematic of models and timing of microCT imaging. (A) One day prior to instillation with LPS mice received microCT imaging. Animals were followed longitudinally with additional scans at day 7 and day 14. Red line depicts the resolving course of disease and inflammation associated with LPS treatment. (B) One day prior to instillation with bleomycin mice received microCT imaging. Animals were followed longitudinally with additional scans at day 21 (3wk) and day 56 (8wk). Blue line depicts the course of resolving disease and fibrosis associated with bleomycin treatment. Additional naïve and fibrotic (day 21 post bleomycin) animals underwent respiration-gated CT imaging (blue text). (C) One day prior to instillation with silica mice received microCT imaging. Animals were followed longitudinally with additional scans at day 56 (8wk) and day 84 (12wk). Green line depicts the course of progressive disease and fibrosis associated with silica treatment. Additional fibrotic (day 56 post silica) animals underwent nitrogen-inflation CT imaging or were fixed and imaged *ex vivo* (green text).
(PDF)

S2 Fig Aerated and tissue volumes from post mortem nitrogen-inflated imaging. Representative transverse sections from mice prior to silica instillation (naïve), and at 8 weeks post-instillation are shown (top row). The aerated lung volume (red) is overlaid with the image (second row), and the extracted aerated lung ROI (third row) are shown. Representative overlay of the tissue volume (red) and image are shown (fourth row), as is the extracted tissue ROI (last row).
(PDF)

S1 Methods (PDF)
