# Supplementary material for: Application-specific approaches to MicroCT for evaluation of mouse models of pulmonary disease
Source: PLoS One. 2023 Feb 9;18(2):e0281452. doi: 10.1371/journal.pone.0281452 (PMC9910664; doi:10.1371/journal.pone.0281452)
Supplement: S1 Fig — (A) One day prior to instillation with LPS mice received microCT imaging. Animals were followed longitudinally with additional scans at day 7 and day 14. Red line depicts the resolving course of disease and inflammation associated with LPS treatment. (B) One day prior to instillation with bleomycin mice received microCT imaging. Animals were followed longitudinally with additional scans at day 21 (3wk) and day 56 (8wk). Blue line depicts the course of resolving disease and fibrosis associated with bleomycin treatment. Additional naïve and fibrotic (day 21 post bleomycin) animals underwent respiration-gated CT imaging (blue text). (C) One day prior to instillation with silica mice received microCT imaging. Animals were followed longitudinally with additional scans at day 56 (8wk) and day 84 (12wk). Green line depicts the course of progressive disease and fibrosis associated with silica treatment. Additional fibrotic (day 56 post silica) animals underwent nitrogen-inflation CT imaging or were fixed and imaged ex vivo (green text). (PDF) [file pone.0281452.s001.pdf]

**Supporting Information**
**Application-specific Approaches to MicroCT for Evaluation of Mouse Models of Pulmonary Disease**

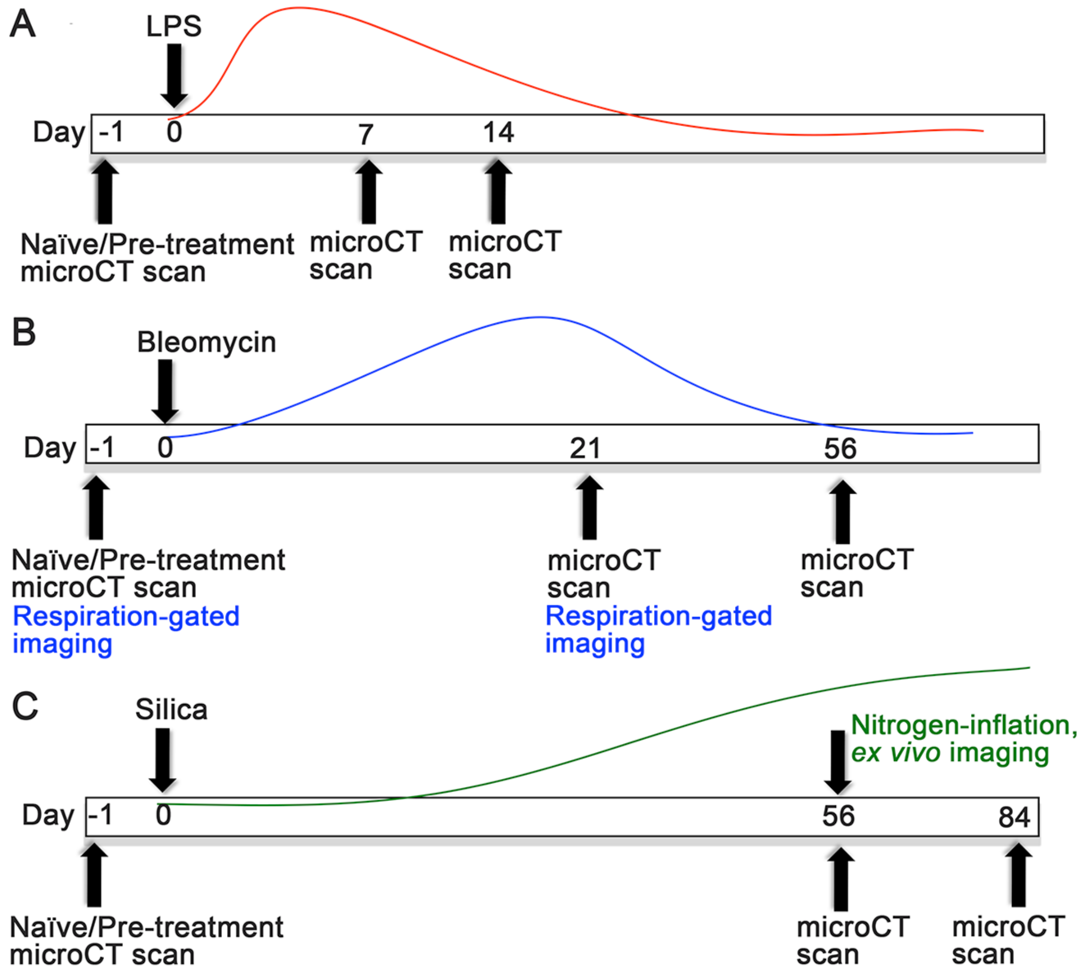


**Supplemental Figure 1. Schematic of models and timing of microCT imaging.** (A) One day prior to instillation with LPS mice received microCT imaging. Animals were followed longitudinally with additional scans at day 7 and day 14. Red line depicts the resolving course of disease and inflammation associated with LPS treatment. (B) One day prior to instillation with bleomycin mice received microCT imaging. Animals were followed longitudinally with additional scans at day 21 (3wk) and day 56 (8wk). Blue line depicts the course of resolving disease and fibrosis associated with bleomycin treatment. Additional naïve and fibrotic (day 21 post bleomycin) animals underwent respiration-gated CT imaging (blue text). (C) One day prior to instillation with silica mice received microCT imaging. Animals were followed longitudinally with additional scans at day 56 (8wk) and day 84 (12wk). Green line depicts the course of progressive disease and fibrosis associated with silica treatment. Additional fibrotic (day 56 post silica) animals underwent nitrogen-inflation CT imaging or were fixed and imaged *ex vivo* (green text).
