# Supplementary material for: Application-specific approaches to MicroCT for evaluation of mouse models of pulmonary disease
Source: PLoS One. 2023 Feb 9;18(2):e0281452. doi: 10.1371/journal.pone.0281452 (PMC9910664; doi:10.1371/journal.pone.0281452)
Supplement: S2 Fig — Representative transverse sections from mice prior to silica instillation (naïve), and at 8 weeks post-instillation are shown (top row). The aerated lung volume (red) is overlaid with the image (second row), and the extracted aerated lung ROI (third row) are shown. Representative overlay of the tissue volume (red) and image are shown (fourth row), as is the extracted tissue ROI (last row). (PDF) [file pone.0281452.s002.pdf]

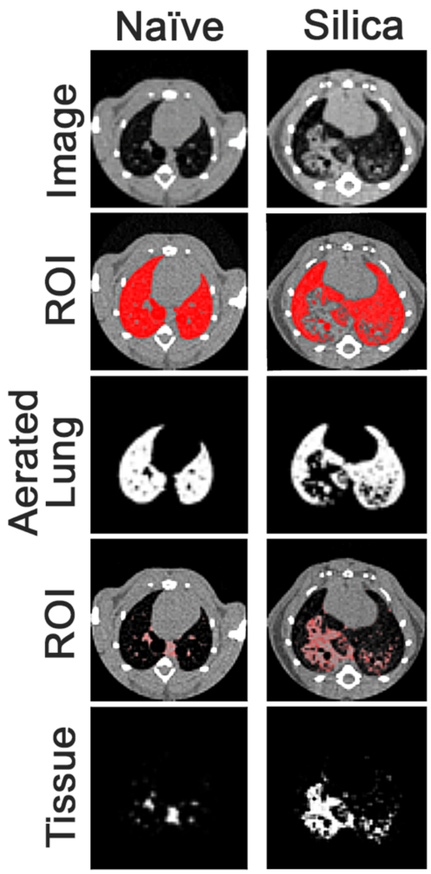

Supplemental Figure 2. Aerated and tissue volumes from post mortem nitrogen-inflated imaging. Representative transverse sections from mice prior to silica instillation (naïve), and at 8 weeks post-instillation are shown (top row). The aerated lung volume (red) is overlaid with the image (second row), and the extracted aerated lung ROI (third row) are shown. Representative overlay of the tissue volume (red) and image are shown (fourth row), as is the extracted tissue ROI (last row).
