# Supplementary material for: Application-specific approaches to MicroCT for evaluation of mouse models of pulmonary disease
Source: PLoS One. 2023 Feb 9;18(2):e0281452. doi: 10.1371/journal.pone.0281452 (PMC9910664; doi:10.1371/journal.pone.0281452)
Supplement: S1 Methods — (PDF) [file pone.0281452.s003.pdf]

S1 Methods

General considerations for analysis and task list modification

1. Consistency in image acquisition is essential for Region of interest (ROI) generation, and all samples must be reconstructed using the same parameters. Appropriate ROIs will combine to become the volume of interest (VOI).
2. All ROIs, whether generated by automation or manually, should be verified by overlaying the ROI on the original reconstructed scan, as well as visualization of the 3D model overlay of air and tissue models (**Suppl. Figure 1**). This provides opportunity to reduce error due to excess excluded or included tissue.
3. ROIs should be developed by blinded investigators to reduce bias, as some interpretation is required when modifying automated ROIs.
4. It may be necessary to incorporate a "pause" at the first comment to verify accuracy of the task list generated aerated lung volume ROI prior to proceeding. Likewise, prior to analysis of the vessels and dense tissue ROI, a "pause" may be required for ROI verification.
5. Additional Despeckle steps may improve the final ROI in cases where less dense body tissue may be included in the task list generated ROI. It is recommended that several trials of the task list be run in this case. Starting with an additional sweep "all except the largest object" or, adding a sweep "the largest object" prior to the Despeckle sweep "all except the largest object" step can improve the result.
6. Depending on reconstruction parameters, particularly histogram settings, adjusting the Thresholding parameters can improve results. For example, in models with extensive inflammation, for the initial aerated ROI, setting the body to 65-255 and then aerated threshold to 0-65 may be beneficial. Or, in samples where dense tissue, rather than inflammation may be of interest, setting a higher tissue threshold (eg. 65- or 75-255) may be beneficial. Note: since the final analysis is largely dependent on the Thresholding parameter it is important that this step be kept consistent among samples within a study.
7. Adjusting the ROI shrink-wrap and/or morphological closing parameters can improve results. This is particularly helpful for samples with large inflamed or fibrotic regions that may not be readily "filled-in" in the task list generated vessels and dense tissue ROI. ROI shrink-wrap with stretch over holes of 4-10 pixels, and Morphological Operations closing of radius 20-40 are recommended starting points.
8. For ex vivo scans, samples can be thresholded for tissue directly. Bitwise operations are performed to set the ROI as a copy of the image, and to set the image=ROI and image. Bitmaps can then be saved, and 3D analysis and modeling performed.

Additional recommended steps for challenging samples

In addition to the considerations above, the following should be considered for disease models with significant inflammation or peripheral tissue (eg. fibrosis, tumor) or tissue adjacent to diaphragm or heart.

1. Modification of task list generated ROI's by hand may be required. Inclusion of the Optional Steps in the tissue segmentation portion of the automated task list can improve results and efficiency by providing a starting ROI with much of the lung outlined. The "ROI for modification" generated by these steps can be loaded and overlaid with the original scan under the ROI tab for modification.
2. When modifying ROI's by hand using shift/right click and ctrl/right click feature, using "interpolate" feature combined with alt/delete function can improve efficiency by interpolating the ROI between revised slices.
3. Alternatively, creation of a VOI by setting the top and bottom of the selection and using the elliptical ROI feature to select lung using the chest wall/ribs as a guide can reduce less dense body tissue and fat from the automated result. Inclusion of sufficient chest wall

tissue in this starting VOI provides a defined border thereby preventing loss of lung tissue from slices where a defined border may otherwise not exist.

4. Comparison to a standard untreated reconstructed scan can assist the investigator in interpreting the boundary of the lung in models where a clear air-filled boundary does not exist either around the periphery of the lung or adjacent to the heart of diaphragm. In cases where clear delineation does not exist, blinding and consistency are particularly important to avoid unintentional bias.
5. Following development of a modified ROI, the sample can be thresholded for tissue, bitwise operations are performed to set ROI=image and ROI, the resulting tissue bitmaps can then be saved and 3D analysis and modeling can be performed.
